# Long-Term Exposure to Air Pollution and Incidence of Venous Thromboembolism in the General Population: A Population-Based Retrospective Cohort Study

**DOI:** 10.3390/jcm11123517

**Published:** 2022-06-19

**Authors:** Jun Gyo Gwon, Sang Ah Lee, Kye-Yeung Park, Se Uk Oh, Joung Soo Kim, Hyun-Min Seo

**Affiliations:** 1 Division of Vascular Surgery, Department of Surgery, Ulsan University College of Medicine, Asan Medical Center, Seoul 05505, Korea; doctorgjg@gmail.com (J.G.G.); ddog94@hanmail.net (S.A.L.); 2 Department of Family Medicine, Hanyang University College of Medicine, Seoul 04763, Korea; kyeyeung@naver.com; 3 Department of Dermatology, College of Medicine, Hanyang University, Hanyang University Guri Hospital, 153, Gyeongchun-ro, Guri-si 11923, Korea; ch001508kr@naver.com (S.U.O.); tuentuen@hanyang.ac.kr (J.S.K.); 4 Hanyang Institute of Bioscience and Biotechnology, Hanyang University, Seoul 04763, Korea

**Keywords:** deep vein thrombosis, pulmonary embolism, particulate matter, disease, national health program, air pollutants

## Abstract

To date, the relationship between air pollutants and venous thromboembolism (VTE) has not been well established. Our aim is to investigate the association between ambient air pollutants and the incidence of VTE using the Korean National Health Insurance Service-National Health Screening Cohort (NHIS-HEALS) database. From 2003 to 2015, 338,616 subjects from the general population not previously diagnosed with VTE were included. The long-term average concentration of air pollutants before diagnosis for each subject was calculated. During the study period, there were 3196 incident cases of VTE. After adjusting for age, gender, economic status, body mass index, physical activity, smoking, alcohol consumption, comorbid diseases, and meteorological variables, the risk of VTE was observed to increase significantly with the long-term average concentration of particulate matter < 10 μm in diameter: PM_10_ (hazard ratio (HR) = 1.064 (95% confidence interval [CI] 1.053–1.074) for 1 μg/m^3^), SO_2_ (HR = 1.118 (95% CI 1.079–1.158) 1 ppb), and O_3_ (HR = 1.039 (95% CI 1.026–1.053) for 1 ppb), respectively. A difference between the date of the health screening and the date of diagnosis of the disease was observed. Long-term exposure to air pollutants including PM_10_, SO_2_, and O_3_ may be an independent risk factor for the development of VTE.

## 1. Introduction

The major air pollutants include CO, SOx, NOx, and fine dust. Although efforts to reduce emissions of these air pollutants are ongoing, air pollution is still a global health problem. Several studies have already shown that air pollutants increase the risk of cardiovascular diseases. A previous study shows that air pollutants can affect the blood clotting process as one of several mechanisms that increase the incidence of cardiovascular diseases [1]. However, thrombi that occur in arteries and veins have different mechanisms of development. In a previous study, clots occurred in the arterial bed, but not in the venous circulation, in experiments using air pollutants [2]. A few studies have analyzed the relationship between air pollutants and venous thromboembolism (VTE); however, these studies had some limitations. The results of these studies are difficult to apply to the current environment because they were conducted before 2010. These studies were conducted in developed countries, and the number of subjects in the studies was too small or air pollution was relatively low [3,4]. A meta-analysis based on these studies confirmed that there was no relationship between air pollution and VTE [5]. The aim of this study is to investigate whether there is a correlation between air pollutants and the occurrence of VTE using real-world data in regions with relatively high air pollution.

## 2. Materials and Methods

### 2.1. Study Design and Database

This retrospective cohort study was conducted on the subjects in the National Health Insurance Service (NHIS)-Health Screening (HEALS) cohort from 2002 to 2015, which comprised random samples representing approximately 0.5 million subjects aged 40–79 years. This encompassed 10% of all health screening participants in Korea [6]. This general health examination program can be applied for by all Korean adults aged 40 years or older every two years. Subjects who received treatment for VTE or comorbidities during the screening period in 2002 were excluded from the analysis. Data regarding demographic characteristics including age, gender, residence, and economic status were collected. Diagnostic codes based on the *International Classification of Diseases, Tenth Revision* (ICD-10), were retrieved. 

### 2.2. Study Subjects and Definition of Clinical Outcomes 

Subjects who had ICD-10 codes of I80, I80.1, I8.02, I80.3, I80.8, I8.09, I82, I82.2, I82.8, I82.9 (deep vein thrombosis), I26, I26.0, and I26.9 (pulmonary embolism) were identified from the NHIS-HEALS. Only the subjects prescribed low-molecular-weight heparin, heparin, warfarin, or new oral anticoagulants were included, in order to improve the accuracy of the diagnosis. The date of disease diagnosis was used as the date of entry for VTE subjects.

### 2.3. Baseline Demographic and Clinical Characteristics

The baseline characteristics of the subjects, including age, gender, economic status, body mass index (BMI), alcohol consumption, smoking status, and exercising status, were analyzed. The smoking status was classified as non-smoker, ex-smoker, or current smoker. Based on the alcohol consumption of the subjects, the drinking status was categorized as non-drinker, social drinker (1–5 times per week), or heavy drinker (≥6 times per week). Physical activity was classified into irregular and regular activities. Economic status, based on insurance payments, was categorized into quintile groups. Comorbidities, including hypertension, diabetes, heart diseases, stroke, and tuberculosis, were identified using a self-reported questionnaire about past diagnoses.

Laboratory test results of blood hemoglobin, serum fasting glucose, cholesterol, high-density lipoprotein (HDL), low-density lipoprotein (LDL), triglyceride (TG), creatinine, aspartate transaminase (AST), and alanine transaminase (ALT) levels were retrieved from the NHIS-HEALS database. BMI ≥ 25 kg/m^2^ was defined as obesity. High blood pressure (BP) was defined as a systolic BP of 120–139 mmHg or a diastolic BP of 80–89 mmHg. Increased serum glucose was defined as a fasting serum blood glucose level ≥ 100 mg/dL. Increased total cholesterol was defined as a level ≥ 200 mg/dL, and increased TG was defined as a level ≥ 150 mg/dL. Decreased HDL was defined as a level ≤ 60 mg/dL and increased LDL was defined as a level ≥ 130 mg/dL. Increased creatinine was defined as a level > 1.5 mg/dL. Anemia was defined as a blood hemoglobin level ≤ 13 g/dL in men and ≤12 g/dL in women. Increased liver enzymes were defined as serum AST level > 40 IU/L or serum ALT level > 35 IU/L. Increased γ-glutamyl transpeptidase was defined as a level > 63 U/L in men and >35 U/L in women. The reference ranges were set according to the criteria of the NHIS-HEALS database.

### 2.4. Measurements of Air Pollutants

Levels of ambient particulate matter < 10 μm in diameter (PM_10_), gaseous air pollution (including SO_2_, NO_2_, CO, and O_3_), temperature, and relative humidity, measured hourly at the sites of the Korean Nationwide Meteorological Observatory by the Korean Department of Environmental Protection, were collected. In order to evaluate the long-term health effects of air pollutants, the nearest monitor was identified for each residence, and the average pollutant concentrations for each study subject were evaluated. To exclude the effect of exposure to air pollutants after VTE diagnosis, the long-term average concentration of air pollutants before diagnosis was calculated for each subject. For subjects who were not diagnosed with VTE, the average concentration of exposure was calculated from the entry date to the end of the follow-up. The geography-based concentration of each air pollutant was measured per hour at the 313 measurement facilities, and 256 residential ZIP codes were matched with the nearest measurement facilities.

### 2.5. Statistical Analyses

We calculated the incident cases of VTE by dividing the number of events by person-years of risk. Based on the Cox proportional hazards model, the incidence rates were compared when adjusted for age, gender, economic status, BMI, physical activity, smoking, alcohol consumption, comorbidities, and meteorological variables (temperature and humidity). The unadjusted hazards ratios were calculated and adjusted model 1 (adjusted for age, gender, income, BMI, physical activity, smoking, alcohol consumption, and meteorological variables) and fully adjusted model 2 (adjusted for age, gender, income, BMI, physical activity, smoking, alcohol consumption, comorbid diseases, and meteorological variables) were additionally performed. The difference was considered statistically significant when the *p*-value was less than 0.05. We analyzed the data using SAS Enterprise Guide 7.13 (SAS Institute, Cary, NC, USA), and plotted the graph with RStudio (version 1.0.136; R Project for Statistical Computing).

## 3. Results

### 3.1. Baseline and Clinical Characteristics of Incident Cases of VTE

The baseline characteristics of the study population are summarized in Table 1.

A total of 338,578 subjects not previously diagnosed with VTE were included in the study. Over a follow-up period of 12 years, 3158 incident cases of VTE were identified. The mean age of the subjects with VTE (56.97 ± 9.97) was statistically significantly higher than that of the subjects without VTE (51.35 ± 8.78) (*p* < 0.001). There were 1650 (52.25%) men and 1508 (47.75%) women who were diagnosed with VTE during the study period. Both the mean BMI and waist circumference showed statistically significantly higher values in the subjects with VTE than in those without VTE, and the proportion of obesity was also significantly higher in the subjects with VTE than in those without VTE. Regarding comorbidities, the prevalence of hypertension, heart diseases, and stroke was significantly higher in the subjects with VTE than in those without VTE (*p* < 0.001, respectively).

### 3.2. Laboratory Abnormalities

Consistent with the results of previous medical history, the proportion of subjects with increased total cholesterol and LDL was higher in the subjects without VTE than in those with VTE (*p* < 0.001, respectively). The proportion of those with anemia and increased serum creatine was significantly higher in the subjects with VTE (*p* < 0.001, respectively).

### 3.3. Physical Activity, Alcohol Consumption, and Smoking Status

The subjects with VTE were less physically active than those without VTE. The mean level of physical activity was significantly lower in the subjects with VTE (564.4 ± 561.3 MET-min/week) than in those without VTE (775.3 ± 530.1 MET-min/week) (*p* < 0.001). In the subjects with VTE, 52.79% showed a metabolic equivalent of task (MET) of less than 500 per week, higher than 35.26% of subjects without VTE; their overall physical activity was also lower than that of the subjects without VTE (*p* < 0.001). Notably, regarding alcohol consumption, the proportion of social drinkers among the subjects without VTE was higher than that among those with VTE, of which 60.69% were non-drinkers (*p* < 0.001). Regarding smoking status, the proportion of non-smokers among the subjects with VTE was significantly higher than that among those without VTE (*p* < 0.001). However, the mean total pack years of smoking was significantly higher in the subjects with VTE (23.63 ± 20.60) than in those without VTE (20.96 ± 16.17) (*p* = 0.004).

### 3.4. Association between the Long-Term Average Concentration of Air Pollutants and the Incidence of VTE

There was a significant association between the incidence of VTE and long-term average concentrations of PM_10_, SO_2_, NO_2_, O_3_, and CO in the unadjusted analysis. After adjusting for age, gender, economic status, BMI, physical activity, smoking, alcohol consumption, blood pressure, comorbidities, laboratory results, and meteorological variables, the risk of VTE was observed to significantly increase with the long-term average concentrations of PM_10_ (HR = 1.026 (95% CI 1.004–1.048 for 1 μg/m^3^)), SO_2_ (HR = 1.088 (95% CI 1.005–1.179) 1 ppb), and O_3_ (HR = 1.074 (95% CI 1.046–1.103) for 1 ppb), respectively (Table 2).

**Figure 1:**
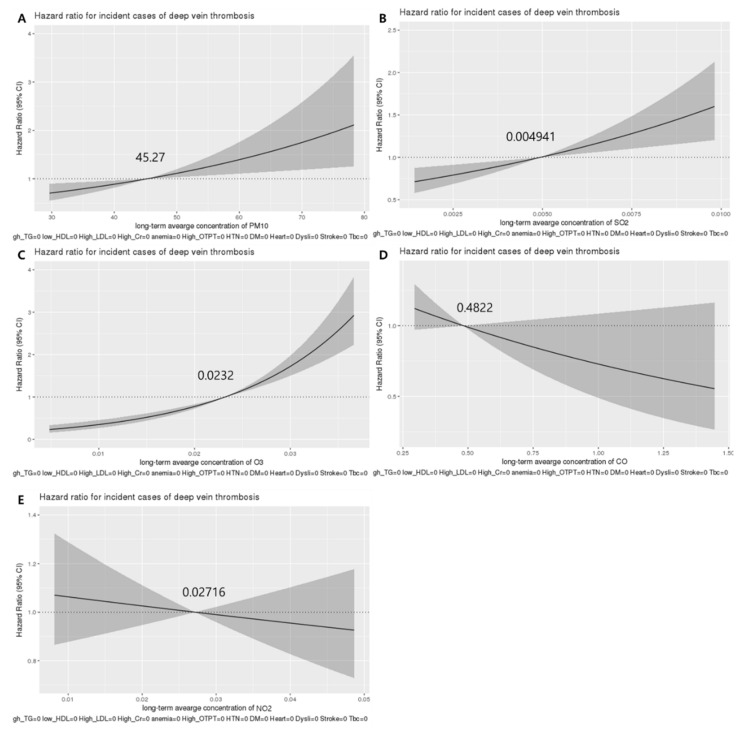
Concentration–response relationships between the incidence of venous thromboembolism and long-term average exposure to air pollutants including PM_10_ (**A**), SO_2_ (**B**), O_3_ (**C**), CO (**D**), and NO_2_ (**E**). The hazard ratio was adjusted for age, gender, economic status, body mass index, physical activity, smoking, alcohol consumption, comorbid diseases, and meteorological variables. CI, confidence interval; O_3_, ozone; PM_10_, particulate matter < 10 μm in diameter; SO_2_, sulfur dioxide.

A Cox proportional hazards model with spline terms for adjusting factors was used. The relationship between the long-term average concentration of each air pollutant and the incidence of VTE was plotted in Figure 1.

The threshold value of the long-term average concentration of each air pollutant with a significant increase in incident VTE risk was 45.27 μg/m^3^ for PM_10_, 0.004941 parts per million (ppm) for SO_2_, and 0.0232 ppm for O_3_.

## 4. Discussion

This present study showed that long-term exposure to PM_10_, SO_2_, and O_3_ was associated with an increased incidence of VTE. However, exposure to CO and NO_2_ did not show significant association with the incidence of VTE. The threshold value of the long-term average concentration of each air pollutant at which the HR for incident VTE risk increased significantly was 45.27 μg/m^3^ for PM_10_, 0.004941 ppm for SO_2_, and 0.0232 ppm for O_3_.

Air pollution is a complex mixture of particulate material and gaseous substances, consisting of solid or liquid substances suspended in the air that vary continuously in chemical composition and size across space and time [7]. Notably, the presence of PM poses more danger to human health than does the presence of other common air pollutants [8]. Indicators describing commonly used health-related PMs refer to the mass concentrations of particles less than 10 μm (PM_10_) in diameter and particles less than 2.5 μm (PM_2.5_) in diameter [7].

In the present study, we aimed to investigate the relationship between air pollutants and the incidence of VTE. Unlike other cardiovascular diseases, there are few studies that have examined the association between air pollution and the risk of VTE. A meta-analysis by Tang et al., which analyzed eight studies, showed that there was no significant association between exposure to major air pollutants and the risk of VTE [5]. However, a systematic review of 11 studies by Franchini et al. presented a possible significant relationship between ambient air pollution and the risk of VTE. Robertson et al. analyzed 74 studies regarding air pollution and thrombosis; there have been too few studies about the effects of gaseous pollutants on thrombosis. A few epidemiological studies suggest that gaseous pollutants, including NO_2_, O_3_, and SO_2_, are associated with thrombosis; however, there are substantial inconsistences between those studies [9]. Thus, the current evidence is too sparse to draw conclusions about the effects of exposure of individual gaseous pollutant on hemostasis. Similarly, distinguishing the effects of individual pollutants in epidemiological studies, such as this present study, remains challenging, especially when correlations between pollutants are high [9]. Accordingly, at present, there is no consensus about the association between air pollutants and VTE. Moreover, each study had a few limitations. First, most existing studies have been conducted in Western countries, where the air pollution levels are much lower than those in Asian countries. According to the WHO database, the PM_2.5_ concentration in the US in 2016 was 7.43 (7.35–7.58) μg/m^3^ and that in Italy was 14.84 (15.56–16.11) μg/m^3^. One the other hand, the PM_2.5_ concentration in the country where this study was conductedwas 26.41 (24.42–29.07) μg/m^3^ in 2016, which is higher than that in Western countries [10]. Second, most of the studies were focused on only PM; Two recent studies by Renzi et al. showed positive associations between both long term and short-term exposure to PM_2.5_ and a risk of VTE [11,12]. However, these findings were confined to the effects of PM_2.5_. Therefore, the effects of gaseous substances such as CO, SO_2_, and O_3_ on VTE is not clear. Third, several studies analyzed VTE hospital admissions as a primary outcome [3,4]. However, according to the European Surgery and Endovascular Surgery guideline for venous thrombosis, outpatient management is recommended for VTE patients. Thus, the incidence of VTE in those studies might have been underestimated. In this present study, we analyzed the association between long-term exposure to air pollutants and VTE occurrence using a relatively large cohort that was derived from a nationwide, general population. Moreover, this study was conducted in cities, where the air pollutant level is higher than that of Western countries, and we analyzed the primary outcome as the diagnosis of VTE using the national data of diagnostic codes, not simply hospital admissions for VTE.

There are a number of possible mechanisms for increased risk of VTE by air pollutants. The relatively well-known mechanisms of increased risk of cardiovascular diseases by air pollutants are divided into two main effects: direct effects and indirect effects [13]. First, the direct effects are mediated by the air pollutants gaining direct entry into the cardiovascular system. Several epidemiological studies have shown that PM exposure shortens the prothrombin time and increases the plasma fibrinogen level. Similarly, an animal study showed that, when an air pollutant is directly instilled into the tracheal space, it may induce a pro-coagulable state by shortening the PT and increasing the PLT count, the V, VII, and X coagulative factors, and fibrinogen [14,15,16,17]. This pro-coagulable state plays important roles in the pathophysiology of VTE. Second, the indirect effects are mediated through pulmonary oxidative stress and inflammatory responses to inhaled pollutants. This chronic indirect effect, with its inflammatory responses, induces a systematic inflammatory state, resulting in the activation of hemostatic pathways, impairing vascular function, and accelerating atherosclerosis [13]. Venous wall inflammation might be the first step in the initiation of venous thrombus formation [18]. During inflammation, the hemostatic balance is disturbed, resulting in an increased production of procoagulant factors and the down-regulation of anticoagulant mechanisms [19]. An animal study found the absence of the PM prothrombotic pathway in IL-6 knockout mice. This result suggests that inflammation plays an important role in the pro-coagulative effects induced by air pollutants [20]. However, the contribution of each individual gaseous air pollutant was not accurately determined and the mechanisms remain unclear.

This present study has some limitations. First, in our study, only outdoor air pollution was analyzed. It is observed that, nowadays, individuals spend most of their time indoors; therefore, the influence of indoor air pollution might be considerable. Second, we cannot exclude the other unadjusted confounding factors. We analyzed the effect of air pollution on VTE with adjustment for age, gender, economic status, BMI, physical activity, smoking, alcohol consumption, blood pressure, comorbidities, laboratory results, and meteorological variables. However, some important confounders (e.g., cancer, statin, antithrombotic agents) were not considered in this study.

## 5. Conclusions

This present study suggests that, in general populations, long-term exposure to PM_10_, SO_2_, and O_3_ is associated with an increased incidence of VTE. Additionally, the thresholds of the long-term average concentrations of each air pollutant at which the HR for incident VTE risk significantly increased were 45.27 μg/m^3^ for PM_10_, 0.004941 parts per million (ppm) for SO_2_, and 0.0232 ppm for O_3_. Our large cohort study may provide strong support for the significant association between air pollution and the occurrence of VTE. There is a need to reduce exposure to air pollution to alter the incidence of VTE as well as other cardiovascular diseases.

## Figures and Tables

**Table 1:** Demographics of subjects with and without venous thromboembolism.

Characteristics	Subjects with VTE (N = 3158)	General Population without VTE (N = 335,420)	*p*-Value
Age, mean ± SD	56.97 ± 9.97	51.35 ± 8.78	<0.001
Gender, N (%)			0.024
Male	1650 (52.25)	182,012 (54.26)	
Female	1508 (47.75)	153,408 (45.74)	
Economic status, N (%)			0.009
0–2 decile (the lowest level)	510 (16.15)	50,039 (14.92)	
3–4 decile	476 (15.07)	44,219 (13.18)	
5–6 decile	429 (13.58)	51,451 (15.34)	
7–8 decile	647 (20.49)	69,750 (20.79)	
9–10 decile (the highest level)	1096 (34.71)	119,961 (35.76)	
Body mass index, mean ± SD	24.36 ± 3.44	23.99 ± 3.02	<0.001
Obesity, N (%) *	1289 (40.83)	116,513 (34.75)	<0.001
Waist circumference, cm	83.88 ± 9.06	82.38 ± 8.46	<0.001
High blood pressure	2385 (74.62)	236,336 (70.47)	<0.001
Systolic blood pressure, mmHg	128.10 ± 16.67	125.60 ± 14.93	<0.001
Diastolic blood pressure, mmHg	78.25 ± 10.81	76.81 ± 9.71	<0.001
Hypertension	641 (52.71)	114,347 (44.46)	<0.001
Diabetes	225 (19.93)	42,706 (18.48)	0.211
Heart diseases	136 (12.35)	16,196 (7.34)	<0.001
Stroke	58 (5.32)	6252 (2.87)	<0.001
Tuberculosis	24 (2.68)	5399 (2.55)	0.679
Increased total cholesterol	1265 (39.63)	146,589 (43.71)	<0.001
Increased triglyceride	762 (28.01)	94,584 (28.71)	0.428
Decreased high-density lipoprotein	1940 (71.27)	230,580 (69.94)	0.132
Increased low-density lipoprotein	782 (28.78)	108,376 (32.99)	<0.001
Increased creatinine	61 (2.24)	4411 (1.34)	<0.001
Anemia	619 (19.39)	41,478 (12.37)	<0.001
Increased liver enzyme	466 (14.6)	53,009 (15.81)	0.063
Increased γ-glutamyl transpeptidase	513 (16.07)	52,084 (15.53)	0.401
Physical activity, min/week			<0.001
<500 MET	795 (52.79)	101660 (35.26)	
500–1000 MET	416 (27.62)	102351 (35.50)	
1000–1500 MET	179 (11.89)	55708 (19.32)	
≥1500 MET	116 (7.70)	28629 (9.93)	
MET, mean ± SD	564.4 ± 561.3	775.3 ± 530.1	<0.001
Alcohol consumption, N (%)			<0.001
Non-drinker	1877 (76.21)	56,545 (40.97)	
Social drinker	449 (18.23)	71,673 (51.93)	
Heavy drinker	137 (5.56)	9789 (7.09)	
Smoking status, N (%)			<0.001
Non-smoker	2168 (70.87)	212,620 (63.61)	
Ex-smoker	448 (14.65)	73,562 (22.01)	
Current smoker	443 (14.48)	48,064 (14.38)	
Pack years	23.63 ± 20.60	20.96 ± 16.17	0.004

VTE, venous thromboembolism; SD, standard deviation. * Defined as body mass index ≥ 25 kg/m^2^. High blood pressure was defined as a more than systolic blood pressure of 139 mmHg or a diastolic BP of 89 mmHg; increased serum glucose was defined as a fasting serum blood glucose level ≥ 100 mg/dL; increased total cholesterol was defined as a level ≥ 200 mg/dL; increased triglyceride was defined as the level ≥ 150 mg/dL; decreased high-density lipoprotein was defined as a level ≤ 60 mg/dL; increased low-density lipoprotein was defined as a level ≥ 130 mg/dL; increased creatinine was defined as a level > 1.5 mg/dL; anemia was defined as a blood hemoglobin level ≤ 13 g/dL in men and ≤ 12 g/dL in women; increased liver enzyme was defined as a serum aspartate transaminase level > 40 IU/L or a serum alanine transaminase level > 35 IU/L; increased γ-glutamyl transpeptidase was defined as a level > 63 U/L in men and > 35 U/L in women.

**Table 2:** Ambient particulate and gaseous air pollution and incidence of venous thromboembolism in the overall general population (*n* = 338,616).

Air Pollutant	HR (95% CI)	*p*-Value	HR (95% CI)	*p*-Value	HR (95% CI)	*p*-Value
	Unadjusted		Adjusted Model 1 *		Adjusted Model 2 **	
PM_10_^†^	1.059 (1.052–1.066)	<0.001	1.024 (1.008–1.040)	0.003	1.026 (1.004–1.048)	0.018
SO_2_^‡^	1.203 (1.178–1.229)	<0.001	1.097 (1.036–1.162)	0.002	1.088 (1.005–1.179)	0.038
NO_2_^‡^	0.993 (0.989–0.997)	<0.001	0.997 (0.986–1.008)	0.587	0.999 (0.984–1.014)	0.892
O_3_^‡^	1.064 (1.058–1.069)	<0.001	1.075 (1.054–1.097)	<0.001	1.074 (1.046–1.103)	<0.001
CO ^§^	0.892 (0.866–0.919)	<0.001	0.947 (0.877–1.022)	0.159	0.949 (0.856–1.053)	0.328

CI, confidence interval; CO, carbon monoxide; HR, hazard ratio; NO_2_, nitrogen dioxide; O_3_, ozone; PM_10_, particulate matter < 10 μm in diameter; ppb, parts per billion; SO_2_, sulfur dioxide. ^†^: by 1 μg/m^3^ increase; ^‡^: by 1 ppb increase; ^§^: by 0.1 ppm increase. *: Adjusted for age, gender, economic status, body mass index, physical activity, smoking, alcohol consumption and meteorological variables. **: Adjusted for age, gender, economic status, body mass index, physical activity, smoking, alcohol consumption, blood pressure, comorbidities, laboratory results, and meteorological variables.

## Data Availability

Data was obtained from NHIS and available with the permission of NHIS.
